# Time to correct the flow of corrected flow time

**DOI:** 10.1186/s13089-017-0076-x

**Published:** 2017-10-04

**Authors:** Igor Barjaktarevic, Alan Chiem, Maxime Cannesson

**Affiliations:** 1 Division of Pulmonary and Critical Care Medicine, David Geffen School of Medicine at University of California, Los Angeles, USA; 2 Department of Emergency Medicine, Olive View-UCLA Medical Center, University of California, Los Angeles, USA; 3 Department of Anesthesiology, David Geffen School of Medicine at University of California, Los Angeles, USA

**Keywords:** Corrected flow time, Carotid corrected flow time, Fluid responsiveness, Shock, Critical care ultrasound

## Abstract

Recently published study of Ma et al. evaluates two relatively novel measures of fluid responsiveness, carotid blood flow and corrected carotid flow time (ccFT). Both measures have been recently quoted as possibly useful, technically simple, and noninvasive dynamic tools in predicting fluid responsiveness. Recently, more research interest has been focused on ccFT and, intrigued by the data presented in this study, we discuss here the impact of the data presented in the paper of Ma et al. to the significance of this metric as a potential tool in the assessment of fluid responsiveness.

Dear Editor,

It was a great pleasure to read about the study of Ma et al. [1] which evaluates two relatively novel measures of fluid responsiveness, carotid blood flow (cBF) and corrected carotid flow time (ccFT, or “corrected CFT” as authors abbreviate). Both measures have been recently quoted as possibly useful, technically simple, and noninvasive dynamic tools in predicting fluid responsiveness [2, 3]. In this study, these metrics are compared to the widely accepted standard of cardiac output (CO) measurement. Of the two measures, cBF has been investigated over past years and remains potentially very valuable metric but may be more prone to measurement error due to variability associated with insonation angle [4]. Recently, more research interest has been focused on ccFT and, intrigued by the data presented in this study, we discuss here the impact of the data presented in the paper of Ma et al. to the significance of this metric as a potential tool in the assessment of fluid responsiveness.

The study of Ma et al. sheds a new light on the role of ccFT and is the first prospective clinical study to compare ccFT with the gold standard of cardiac output measurement, right heart catheterization (RHC). This study evaluated patients undergoing diagnostic RHC who were subjected to a passive leg raise maneuver (PLR) which mimics a fluid bolus and has been used to assess fluid responsiveness in shock [5]. Unfortunately, as there were no differences in CO with PLR, the authors evaluated the correlation of baseline ccFT with baseline CO measured by RHC and showed that ccFT measurements using three waveforms correlated significantly, but weakly, with CO. This is a novel finding but its relevance in determining whether the change in ccFT may help in detecting fluid responsiveness is probably very limited. In order to better comprehend the actual usefulness of corrected flow time, it is important to recognize both the limits of this study and the metric itself.

In this study, ccFT did not correlate well with cardiac output which is not surprising. Flow time is a proxy for left ventricular ejection time, which has classically been considered to represent a static hemodynamic measure, along with other “static” preload indices [6, 7], and thus has been shown not to be adequate measure to predict the change in cardiac output induced by fluid bolus. Nevertheless, instead of looking at correlation of absolute values of ccFT with CO values, it may be the *changes* in this measures that may correlate better. Several studies have looked at the change of ccFT (∆ccFT) as a potential tool in the process of evaluation of fluid responsiveness and found that change in duration of ccFT in dehydrated patients receiving intravenous fluid resuscitation [2, 8, 9] or patients donating blood [10] may be helpful in fluid management in hypotensive patients. Physiologically, with steady inotropy and fixed outflow surface area, the change in the relative duration of systole should correlate with the stroke volume change [8]. Unlike mean arterial blood pressure or heart rate, ccFT may be influenced even by small changes in left ventricle preload, as indicated by significant differences between pre- and post-passive leg-raise maneuver as shown in a study with normal volunteers [11]. Practically, the change in flow time (Ventricular Ejection Time, VET) has been used as in algorithms of noninvasive cardiac output monitors (NICOM, Cheetah medical, Newton Center, MA) [12].

There are several methodological concerns worth bringing up in order to separate a moderate correlation of average ccFT and cardiac output presented in the study of Ma et al. and possible usefulness of the change in ccFT in fluid resuscitation of patients in shock. Patients undergoing RHC who were evaluated in the study of Ma et al. may have very little in common with the population of fluid under-resuscitated patients in shock who are usually evaluated with PLR and where the role of ccFT has been recently discussed as possibly relevant [6]. The cohort selection and methodology raise concern that results of this study need to be interpreted with a caution when discussing usefulness of ccFT. None of the patients in studied cohort was hypotensive or was considered for fluid administration. Large portion of patients had significant cardiac disease which could impact accuracy of both thermodilution-based cardiac output measurement and the duration of ccFT [13, 14]. The lack of more clinical information about patients who participated in the study and had cardiac disease (basic echocardiographic features of patients, filling pressures routinely obtained during RHC or indication for RHC) further limit the strength of any conclusion about the relationship between ccFT and CO.

From the technical perspective, an interesting idea presented in the study of Ma et al. is an attempt to avoid the selection bias by averaging ccFT values of multiple (three) heart beats instead of taking a single value. Understanding the impact of breathing on cardiac rhythm, this concept is valid and important but the method may require further optimization as averaging should probably be taken in the context of respiratory cycle rather than randomly selecting three beats.

There is a common agreement that the dynamic parameters have become a gold standard in fluid responsiveness assessment [15], but the search for an ideal test to predict fluid responsiveness continues. Determining fluid responsiveness in the future more precisely may need more than focusing on a single parameter but rather a set of dynamic parameters that could be used as a part of more comprehensive, composite metrics. The assessment of change of ccFT deserves more research as it is a simple and easily obtained metric that may be used as a part of more comprehensive, composite measures, possibly along with carotid blood flow (cBF) to better determine fluid responsiveness in shock [7].

## Abbreviations

∆ccFT: change in carotid corrected flow time
ccFT: carotid corrected flow time
CFT: carotid flow time
cBF: carotid blood flow
CO: cardiac output
LV: left ventricle
RHC: right heart catheterization
PLR: passive leg raise
VET: ventricular ejection time
